# An Immunosensor for the Detection of ULBP2 Biomarker

**DOI:** 10.3390/mi11060568

**Published:** 2020-06-03

**Authors:** Wen-Chi Yang, Su-Yu Liao, Thien Luan Phan, Nguyen Van Hieu, Pei-Yi Chu, Chin-Chang Yi, Hsing-Ju Wu, Kang-Ming Chang, Congo Tak-Shing Ching

**Affiliations:** 1 Department of Pathology, Show Chwan Memorial Hospital, Changhua City 500, Taiwan; ag57520@hotmail.com (W.-C.Y.); chu.peiyi@msa.hinet.net (P.-Y.C.); 2 Department of Electrical Engineering, National Chi Nan University, Nantou County 545, Taiwan; suyu@ncnu.edu.tw; 3 Graduate Institute of Biomedical Engineering, National Chung Hsing University, Taichung City 402, Taiwan; phanluan1101@gmail.com; 4 Department of Physics and Electronic Engineering, University of Science (Vietnam National University of Hochiminh City), Hochiminh City 700000, Vietnam; nvhieu@hcmus.edu.vn; 5 School of Medicine, College of Medicine, Fu Jen Catholic University, New Taipei City 24205, Taiwan; 6 National Institute of Cancer Research, National Health Research Institutes, Tainan 70456, Taiwan; 7 Department of Surgery, Taichung Armed Forces General Hospital, Taichung City 41152, Taiwan; sacrify0524@gmail.com; 8 Research Assistant Center, Show Chwan Memorial Hospital, Changhua City 500, Taiwan; hildacmuh@gmail.com; 9 Department of Medical Research, Chang Bing Show Chwan Memorial Hospital, Changhua 505, Taiwan; 10 Department of Photonics and Communication Engineering, Asia University, Taichung 41354, Taiwan; 11 Department of Medical Research, China Medical University Hospital, China Medical University, Taichung 40402, Taiwan

**Keywords:** pancreatic cancer, ULBP2, screen-printed, immunosensor, nanoparticles, array configuration

## Abstract

Pancreatic cancer (PC) is a global health problem that features a very high mortality rate. The UL16 binding protein 2 (ULBP2) is a new biomarker for PC detection. This study develops a simple, reliable, and inexpensive immunosensor for the detection of the ULBP2 antigen while also investigating the effects of an array configuration of connected sensors and zinc oxide (ZnO) nanoparticles on the immunosensor’s sensitivity. The ULBP2 antibody was immobilized onto the screen-printed carbon electrode (SPCE) surfaces of three different sensors: a simple SPCE (ULBP2-SPCE); an SPCE array, which is a series of identical SPCE connected to each other at different arrangements of rows and columns (ULBP2-SPCE-1x2 and ULBP2-SPCE-1x3); and an SPCE combined with ZnO nanoparticles (ULBP2-ZnO/SPCE). Impedance spectrum measurements for the immunosensors to ULBP2 antigen were conducted and compared. According to the result, the array configurations (ULBP2-SPCE-1x2 and ULBP2-SPCE-1x3) show an improvement of sensitivity compared to the ULBP2-SPCE alone, but the improvement is not as significant as that of the ULBP2-ZnO/SPCE configuration (ULBP2-ZnO/SPCE > ULBP2-SPCE: 18 times larger). The ULBP2-ZnO/SPCE immunosensor has a low limit of detection (1 pg/mL) and a high sensitivity (332.2 Ω/Log(pg/mL)), excellent linearity (R^2^ = 0.98), good repeatability (coefficients of variation = 5.03%), and is stable in long-term storage (retaining 95% activity after 28 days storage). In an array configuration, the immunosensor has an increased signal-to-noise ratio (ULBP2-SPCE-1x3 > ULBP2-SPCE: 1.5-fold) and sensitivity (ULBP2-SPCE-1x3 > ULBP2-SPCE: 2.6-fold). In conclusion, either the modification with ZnO nanoparticles onto the sensor or the use of an array configuration of sensors can enhance the immunosensor’s sensitivity. In this study, the best immunosensor for detecting ULBP2 antigens is the ULBP2-ZnO/SPCE immunosensor.

## 1. Introduction

Pancreatic cancer (PC) is a serious public health problem worldwide that has a very high mortality rate [1]. PC is respectively the fourth and eighth most common cause of cancer death in the United States [2,3] and Taiwan [4]. In Taiwan, PC accounts for 11.6% of deaths from cancer, and it is estimated that 8.1 out of 100,000 people die because of PC [4]. Deaths from PC have also increased most (17.7%) in the standardized mortality rate for the top ten cancers in terms of statistics that have been reported since 2004 [4]. In the early stages, PC is commonly unobservable, and its symptoms are vague and nonspecific. Therefore, PC is normally diagnosed in the late stages. This results in a heavy burden for national health insurance.

Clinically, PC is screened by measuring the index of cancer antigen 19-9 (CA 19-9) [5] and the carcinoembryonic antigen (CEA) [6], together with noninvasive screening, including abdominal ultrasound imaging and abdominal computed tomography imaging. However, both abdominal ultrasound imaging [7,8] and abdominal computed tomography imaging [9,10] have limitations in terms of their low sensitivity.

A review study (1323 patients in total) that analyzed 13 published journal articles showed that the median sensitivity (range: 40%–92%) and specificity (range: 59%–90%) of CEAs to PC are low, at about 54% and 79%, respectively [11]; here, the sensitivity of a biomarker for its antigen detection is defined as the probability of a correct diagnoses of “positive” cases, and the specificity is the probability of correct diagnosis of the “negative” cases of its antigen. For a biomarker to be of clinical value, it needs to have a sensitivity of at least 90%, and the specificity should also be 90% or more for the detection of its antigen [12]. Studies also show that the CEA has a relatively low sensitivity for PC patients who do not demonstrate cancer metastasis [13,14]. Therefore, the CEA cannot be used alone to detect PC. It must be used in combination with other biomarkers for a PC test [5]. The review study (2283 patients in total) also showed that the respective median sensitivity (range: 70%–90%) and specificity (range: 68%–91%) of CA 19-9 (an FDA approved biomarker for PC) to PC is about 79% and 82%, respectively [11]. CA 19-9 has a low sensitivity to PC and can give a false-positive result, so it is not an effective screening test for PC [15,16]. Stomach cancer [17] and other diseases [18,19], such as liver cirrhosis or cholangitis, can also produce an increase in the level of CA 19-9. If a patient is suspected to be suffering from PC, further examination using endoscopic retrograde cholangiopancreatography (ERCP) may be required [20]. However, these screening tests are complicated and time-consuming and must be performed at the hospital and not at home. This means that it is difficult to discover PC in its early stages.

Recent research shows that the UL16 binding protein 2 (ULBP2) is a biomarker for PC, with about 84% sensitivity and 74% specificity for the detection of PC [16]. A receiver operating characteristic (ROC) curve analysis shows that the ULBP2 distinguishes patients with early-stage PC from normal healthy subjects better than CA 19-9 can [16]. In the past twenty years, several studies have reported the use of either exceptional antibodies or a bead-based immunoassay, together with a flow cytometer, to measure ULBP2 [16,21,22,23]. One study reported the development of a high-throughput biosensor for the determination of ULBP2 antigens that uses metal-enhanced fluorescence and harmonic intensity-modulated fluorescence [24]. Although this high-throughput biosensor is accurate and sensitive for the detection of ULBP2, with a limit of detection (LOD) of about 16 pg/mL, its signal detection method uses an expensive and sophisticated optical laser, so it cannot be used for PC screening at home. Aside from the ULBP2 biosensors, sandwich-type electrochemical immunosensors have also been reported for the detection of the CA 19-9 antigen [25,26]. Table 1 summarizes the performances of some electrochemical biosensors used for the detection of PC biomarkers. Among the electrochemical biosensors listed, electrochemical impedance spectroscopy (EIS) is the most used technique for electrochemical biosensing of PC biomarkers because it is label free.

In this paper, we discuss the sensitivity of the developed biosensor, which should not be confused with the definitions of sensitivity as well as the specificity of a biomarker to its antigen mentioned above. The sensitivity of sensors is defined as the slope of the sensor’s transfer function, which is the variation of the output signal as a function of the input signal. For our biosensor, the input signal is the amount of concentration of analyte present in the measuring medium, and the output signal is the impedance response at different frequencies using the EIS technique. The reported limit of detection (LOD) is the lowest concentration of an analyte in a sample that can be detected by the biosensor, and it was calculated using the following equation: LOD = 3$S_{yx}/b$, where $S_{yx}$ is the standard deviation of the response, and b is the slope of the linear regression line [27,28,29].

To summarize, CA 19-9 has a low sensitivity to PC [15,16], and ULBP2 is more sensitive than CA 19-9 to PC [16], so this study develops a simple, reliable, and inexpensive immunosensor for the detection of the ULBP2 antigen by applying the EIS technique. This study also investigates the effects of array configuration and zinc oxide (ZnO) nanoparticles on the immunosensor’s sensitivity.

## 2. Materials and Methods

### 2.1. Chemicals and Reagents

Glutaraldehyde, bovine serum albumin (BSA), phosphate-buffered saline (PBS), and ZnO nanoparticles (20 nm in diameter) were purchased from Sigma Chemical (St Louis, MO, USA). The ULBP2 antigen and antibody were purchased from R&D Systems (Taiwan). Epoxy (EPO-TEK^®^ 509FM-1) was purchased from Epoxy Technology (Billerica, MA, USA). Graphite and silver pastes were purchased from Advanced Conductive Materials (Atascadero, CA, USA). Polyethylene terephthalate (PET) thin film was purchased from 3M. The Millipore Milli-Q UFplus System (Bedford, MA, USA) was used to generate deionized water (resistivity ≥ 18 MΩcm), which was used for all preparations. All chemicals and reagents are commercially available and were used with no further purification.

### 2.2. Equipment

A screen printing machine (Electric Screen Printer AT-45PA, ATMA Champ Ent. Corp., Taoyuan, Taiwan) was used to fabricate the sensor substrate. An impedance analyzer (Precision Impedance Analyzer WK6420C, Wayne Kerr Electronics Ltd., London, UK) was used for impedance (Z) spectrum measurements of the immunosensor.

### 2.3. Fabrication of the Screen-Printed Carbon Electrode (SPCE)

**Figure 1:**
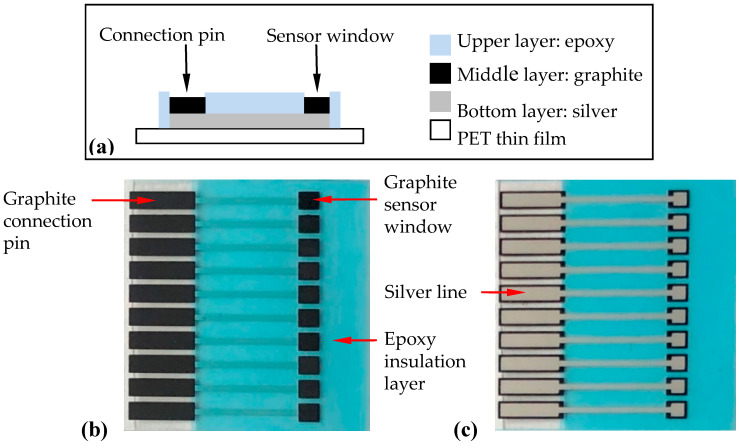
Fabrication of the screen-printed carbon electrode (SPCE) by screen printing. (**a**) Schematic side-view diagram of the SPCE, (**b**) a top-view photo of the SPCE, and (**c**) a bottom-view photo of the SPCE. The substrate was 28 mm × 28 mm while the sensor window’s area was 2 mm × 2 mm.

The SPCE was constructed by screen printing 3 layers onto a PET thin film [34,35] (Figure 1). The bottom layer uses silver as signal conduction lines. The middle layer has graphite pads that form connection pins and sensor window areas for substance (e.g., antibody, nanoparticles) immobilization. The upper layer contains epoxy insulation to insulate protected areas and to form a testing well. After fabrication, the SPCE was composed of an array of ten carbon working electrodes.

### 2.4. Immobilization of ULBP2 Antibody onto SPCE to Form ULBP2-SPCE Immunosensor

**Figure 2:**
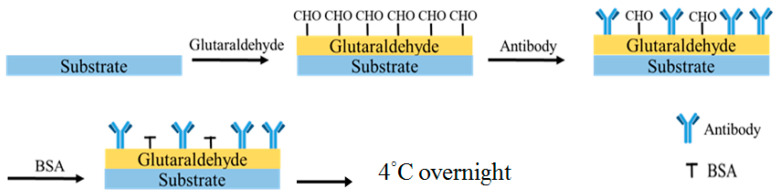
Procedure for the immobilization of the UL16 binding protein 2 (ULBP2) antibody onto a sensor window by drop-coating.

The ULBP2 antibody was immobilized onto the SPCE’s sensor window by drop-coating (Figure 2). Glutaraldehyde (1 μL, 2.5%) was pipetted into the sensor window and one minute later, the ULBP2 antibody (1 μL) was pipetted onto the same sensor window. BSA (0.1 M, 1 μL) was then immediately pipetted onto the same sensor window. Finally, the ULBP2-SPCE immunosensor was allowed to cross-link overnight in the dark at 4 °C.

### 2.5. Immobilization of ULBP2 Antibody and ZnO Nanoparticles onto SPCE to Form ULBP2-ZnO/SPCE Immunosensor

The ULBP2-ZnO/SPCE immunosensor was fabricated by the drop-coating of a mixture (1 µL) of glutaraldehyde (2.5%) and ZnO nanoparticles (0.2 mg/100 mL) into the SPCE’s sensor window. One minute later, the ULBP2 antibody (1 μL) was pipetted onto the same sensor window, followed by BSA (0.1 M, 1 μL). Finally, the ULBP2-ZnO/SPCE immunosensor was allowed to cross-link overnight in the dark at 4 °C.

### 2.6. Construction of ULBP2-SPCE in Array Configurations to Form ULBP2-SPCE-1 × 2 and ULBP2-SPCE-1 × 3 Immunosensors

**Figure 3:**
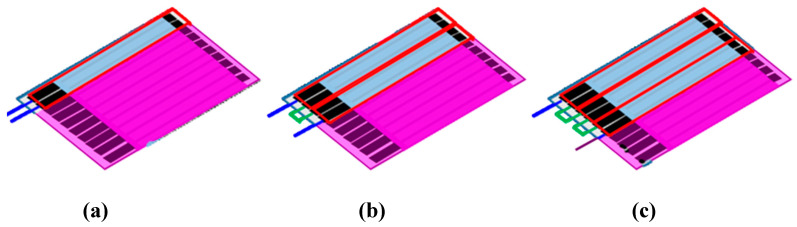
ULBP2-SPCEs in array configuration: Each red rectangle represents each of the identical ULBP2-SPCEs. The blue lines are connected to an impedance analyzer, and the green lines connect identical ULBP2-SPCEs in series: (**a**) 1 × 1 array configuration (i.e., ULBP2-SPCE), (**b**) 1 × 2 array configuration (i.e., ULBP2-SPCE-1 × 2), and (**c**) 1 × 3 array configuration (ULBP2-SPCE-1 × 3).

ULBP2-SPCEs are placed in an array configuration by connecting several identical pairs of ULBP2-SPCEs in series (Figure 3). A single ULBP2-SPCE sensor consists of 2 electrodes in a 1 × 1 (rows × columns) array configuration (Figure 3a). The 1 × 2 array configuration of the ULBP2 sensors, the ULBP2-SPCE-1 × 2 (Figure 3b), connected 2 ULBP2-SPCEs in series, whereas the 1 × 3 array configuration, the ULBP2-SPCE-1 × 3 (Figure 3c), uses 3.

### 2.7. Impedance Spectrum Measurement for the Immunosensors to ULBP2 Antigen

**Figure 4:**
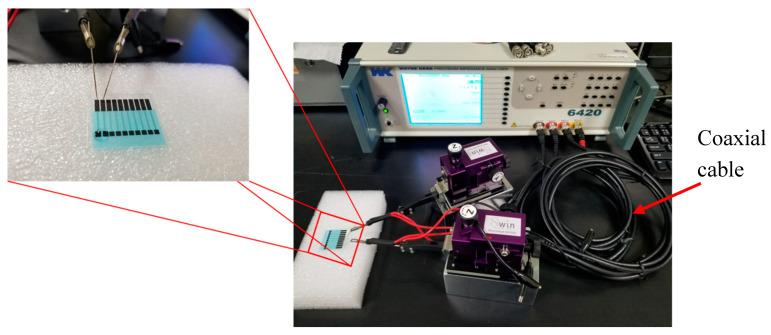
Impedance spectrum measurement experimental setup.

The impedance spectrum of the sample was measured at 25–25.5 °C and was kept constant throughout the measurement. The respective range of measurement frequency and the amplitude of the perturbing wave is 1 kHz–10 MHz and 100 mV. Figure 4 shows the impedance spectrum measurement setup. The experiment involves ten stages:

(a) Connect the immunosensor (ULBP2-SPCE, ULBP2-ZnO/SPCE, ULBP2-SPCE-1 × 2, or ULBP2-SPCE-1 × 3) electrodes to the impedance analyzer probes.
(b) Pipette PBS (25 mM, pH 7.0) onto the sensor windows of the immunosensor and wait for 1 min.
(c) Measure the Z spectra for the PBS and denote the measurement as Z_PBS_.
(d) Remove the PBS.
(e) Pipette 1 μL of the ULBP2 antigen (0.1, 1, 10, 100, 1000 pg/mL) onto each sensor window of the immunosensor and wait for 90 min, which is required for cross-linking.
(f) Rinse the immunosensor gently with fresh PBS (25 mM, pH 7.0) 5 times to remove the ULBP2 antigen that is not cross-linked.
(g) Pipette PBS (25 mM, pH 7.0) onto the sensor windows of the immunosensor and wait for 1 min.
(h) Measure the Z spectra again and denote the measurement as Z_ULBP2_ spectra for the ULBP2.
(i) Calculate the impedance response (ΔZ) of the immunosensor to the ULBP2 antigen by subtracting Z_ULBP2_ from Z_PBS_ (i.e., ΔZ = Z_ULBP2_ – Z_PBS_).
(j) Use a new immunosensor and repeat steps (a) to (i) using different concentrations of the ULBP2 antigen (0.1, 1, 10, 100, 1000 pg/mL).

### 2.8. Evaluation of the Immunosensors

Several tests were conducted to evaluate the performance of the immunosensors, including an optimal measuring frequency test, a linearity test, a sensitivity test, repeatability tests, a life-span test as well as a test to determine the effects of the array configuration and ZnO nanoparticles on the sensor’s sensitivity.

## 3. Results and Discussion

### 3.1. Evaluation of the SPCE and SPCE Coated with a Mixture of ZnO Nanoparticles and Glutaraldehyde

**Figure 5:**
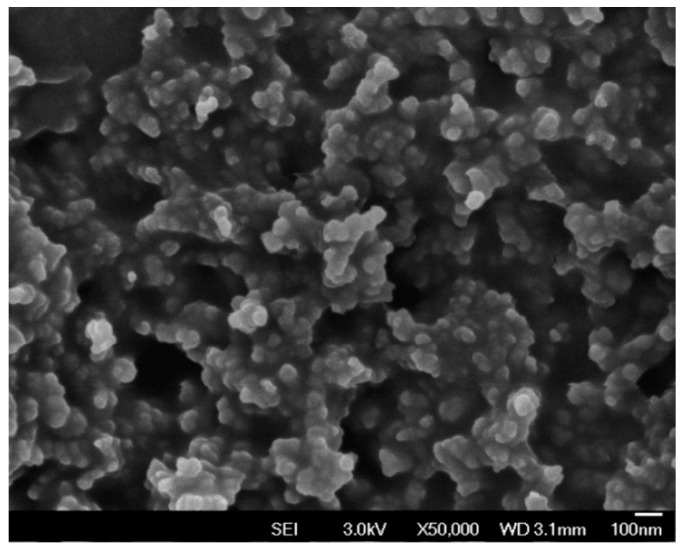
FE-SEM image of the graphite sensor window surface of a SPCE (without ZnO).

**Figure 6:**
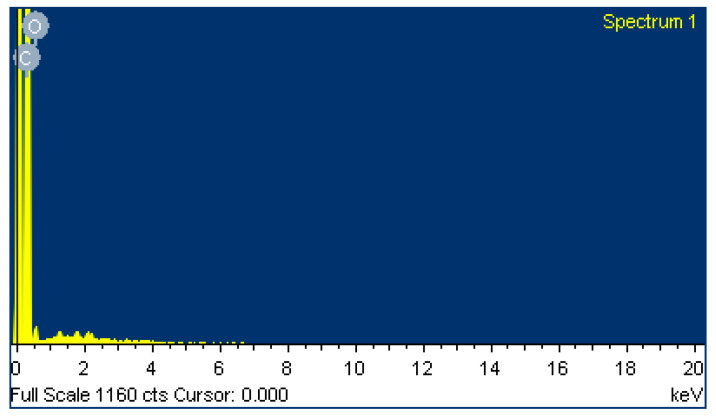
EDS of the graphite sensor window surface for the SPCE.

A field emission scanning electron microscope (FE-SEM) and an energy dispersive spectrometer (EDS) were used to evaluate the graphite sensor window surface of the SPCE. The FE-SEM image (Figure 5) shows that the SPCE has a rough surface with carbon nanoparticles (~50 nm) that are uniformly distributed. Data from the EDS shows that the SPCE is purely fabricated with 94% carbon and 6% oxygen (Table 2 and Figure 6).

**Figure 7:**
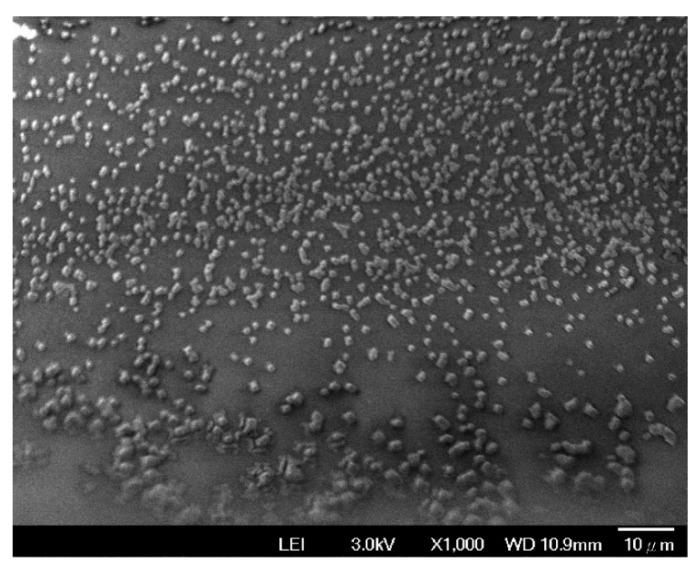
FE-SEM image of the SPCE coated with the mixture of ZnO nanoparticles and glutaraldehyde.

The FE-SEM was also used to evaluate the SPCE coated with the mixture of ZnO nanoparticles and glutaraldehyde. The FE-SEM image (Figure 7) shows that the SPCE is evenly covered with ZnO nanoparticles.

### 3.2. The Optimal Measurement Frequency, Linearity, and Sensitivity of the ULBP2-SPCE and ULBP2-ZnO/SPCE Immunosensors

**Figure 8:**
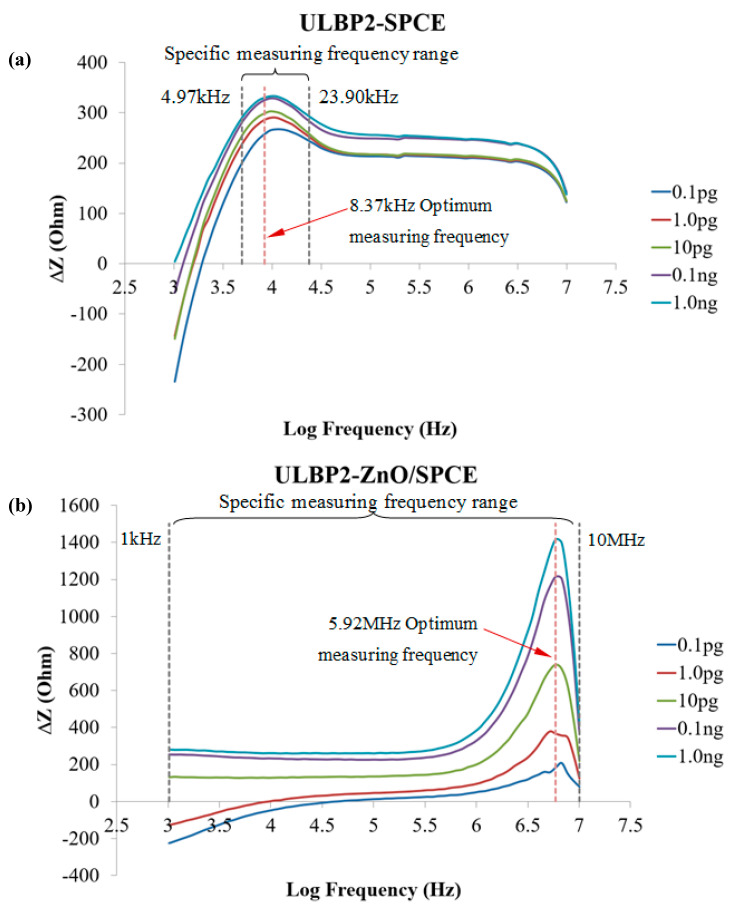
The spectrum for the impedance response of the immunosensors to the ULBP2 antigen at various concentrations: (**a**) ULBP2-SPCE immunosensor: There is an optimal measurement frequency (8.37 kHz) and a specific measurement frequency range (4.95–23.90 kHz). (**b**) ULBP2-ZnO/SPCE immunosensor: There is an optimal measurement frequency (5.92 MHz) and a specific measurement frequency range (1 kHz–10 MHz). Remark: R^2^ is the coefficient of determination between the immunosensor impedance response and the logarithmic concentration of the ULBP2 antigen.

Figure 8a,b shows the impedance response in the frequency spectrum between 1kHz and 10MHz of the ULBP2-SPCE and ULBP2-ZnO/SPCE immunosensors to various concentrations of the ULBP2 antigen, respectively. There is a specific measurement frequency range (ULBP2-SPCE: 4.95–23.90 kHz; ULBP2-ZnO/SPCE: 1kHz–10MHz) within which the measurement at each frequency shows an excellent linear response, namely that it is the frequency range in which the coefficient of determination (R^2^) between the impedance response of the immunosensor and the logarithmic concentration of the ULBP2 antigen greater than 0.9. The optimal measuring frequencies, 8.37 kHz for ULBP2-SPCE and 5.92 MHz for ULBP2-ZnO/SPCE, have the maximum sensitivity, determined by the slope of the fitting equation.

**Figure 9:**
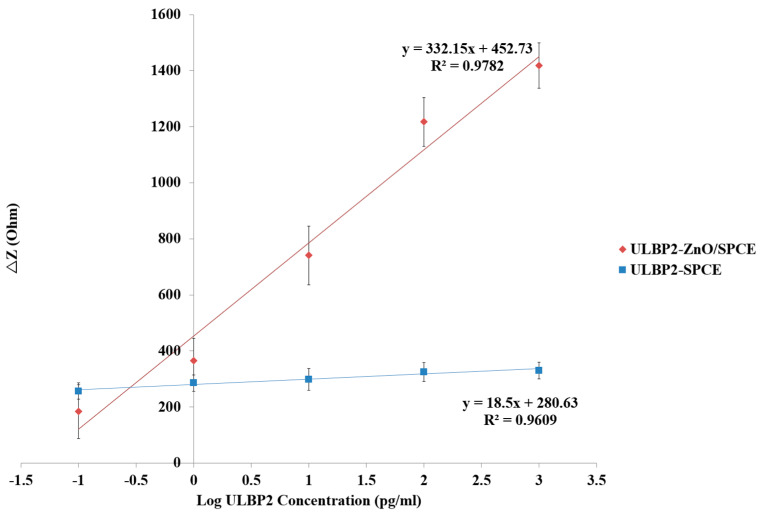
The fitting equations for the transfer characteristics of the ULBP2-SPCE and ULBP2-ZnO/SPCE immunosensors determined at their optimal measurement frequencies (ULBP2-SPCE: 8.37 kHz; ULBP2-ZnO/SPCE: 5.92 MHz).

Figure 9 shows the linear regression line for the ULBP2-SPCE and ULBP2-ZnO/SPCE immunosensors for the measurement of the ULBP2 antigen at their optimal measurement frequencies of 8.37 kHz and 5.92 MHz, respectively. For the ULBP2-SPCE immunosensor, there is an excellent (R^2^ = 0.96) linear relationship between the impedance response of the immunosensor and the logarithmic concentration of the ULBP2 antigen, with a maximum sensitivity of 18.5 Ω/Log (pg/mL) and a limit of detection (LOD) of 4.7 pg/mL. For the ULBP2-ZnO/SPCE immunosensor, an excellent (R^2^ = 0.98) linear relationship is found between the impedance response of the immunosensor and the logarithmic concentration of the ULBP2 antigen, with a maximum sensitivity of 332.2 Ω/Log (pg/mL) and a LOD of 1 pg/mL.

Results show that the ULBP2-ZnO/SPCE immunosensor has a statistically significant (independent *t*-test: *p* value < 0.001) and higher (about 18-fold) sensitivity than that of the ULBP2-SPCE immunosensor. This is because of the large surface area to volume ratio of ZnO nanoparticles, and the surface of ZnO nanoparticles can facilitate the antibody immobilization [36].

### 3.3. Repeatability and Life-Span Tests of the ULBP2-SPCE and ULBP2-ZnO/SPCE Immunosensors

Tests were performed to evaluate the measurement repeatability of the ULBP2-SPCE and ULBP2-ZnO/SPCE immunosensors for the determination of the ULBP2 antigen (Table 3). Five different batches of ULBP2-SPCE and ULBP2-ZnO/SPCE immunosensors were used to detect the ULBP2 antigen under the same conditions. The results of the repeatability test confirm that both ULBP2-SPCE and ULBP2-ZnO/SPCE immunosensors detect the concentrations of the ULBP2 antigen, with a coefficient of variation of 4.55% and 5.03%, respectively. An intraclass correlation coefficient (ICC) is used for the statistical analysis of the test. For many clinical measurements, ICC values must exceed 0.9 to ensure reasonable validity and repeatability [37]. This test confirms that both ULBP2-SPCE and ULBP2-ZnO/SPCE immunosensors have excellent repeatability, with respective ICC(3,k) and ICC(2,k) values of 0.91 and 0.90 for ULBP2-SPCE as well as 0.90 and 0.88 for ULBP2-ZnO/SPCE (Table 3).

**Figure 10:**
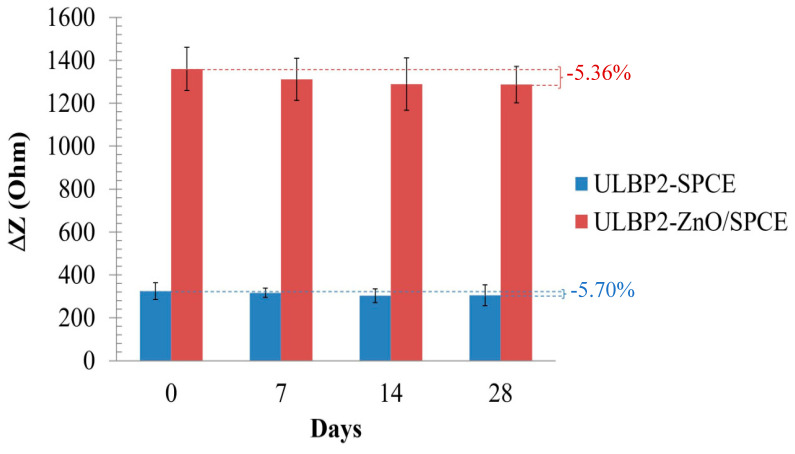
The life-span test for the ULBP2-SPCE and ULBP2-ZnO/SPCE immunosensors.

Figure 10 shows the storage stability of the ULBP2-SPCE and ULBP2-ZnO/SPCE immunosensors at 4 °C in dark and dry conditions for 28 days. The impedance response of both immunosensors is about 95% of its initial value, which shows that both immunosensors have good long-term stability. This may be because of the antibody immobilization technique that is used in this study. Glutaraldehyde and BSA are used to cross-link the ULBP2 antibody onto the sensor. This combination produces a good microenvironment inside the cross-linked structure. ULBP2 antibody that is immobilized at such microenvironment maintains its functional activity.

### 3.4. The Effect of Array Configurations

**Figure 11:**
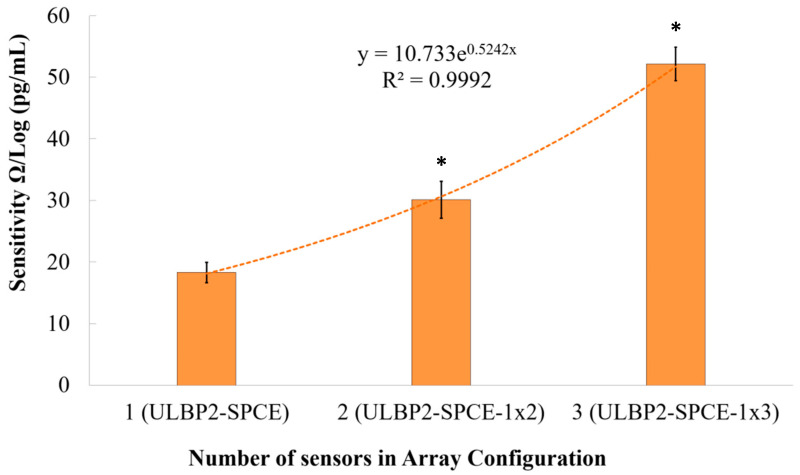
The effect of an array configuration on the immunosensor’s sensitivity. Here, * represents a statistically significant (*p* value < 0.05) difference between ULBP2-SPCE (i.e., 1 × 1 array) and other array configurations, such as ULBP2-SPCE-1 × 2 (i.e., 1 × 2 array) and ULBP2-SPCE-1 × 3 (i.e., 1 × 3 array).

The array configuration setup helps examine the effect of increasing the sensor window area and the effect of connecting the sensors in series on impedance changes. As can be seen from Figure 11, there is an exponential relationship (R^2^ = 0.999) between the immunosensor’s sensitivity and the number of sensors connected in the array configuration. It is seen that the immunosensor’s sensitivity increases exponentially as the array configuration increases in size. This exponential increase means that there is a limit to the amount by which the array configuration can be used to increase the immunosensor’s sensitivity.

Results show that the ULBP2-SPCE-1 × 3 immunosensor has a statistically significant (independent *t*-test: *p* value < 0.05) and higher sensitivity than the ULBP2-SPCE-1 × 2 (about 1.7-fold) and ULBP2-SPCE (about 2.6-fold) immunosensors, while the ULBP2-SPCE-1 × 2 immunosensor also has a statistically significant (independent *t*-test: *p* value < 0.05) and higher sensitivity than the ULBP2-SPCE (about 1.6-fold).

**Figure 12:**
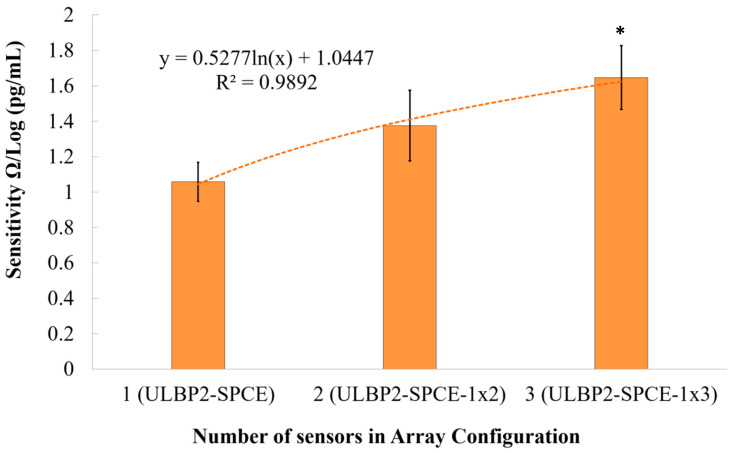
The effect of an array configuration on the immunosensor’s signal-to-noise ratio (SNR). Here, * represents a statistically significant (*p* value <0.05) difference between the ULBP2-SPCE (i.e., 1 × 1 array) and ULBP2-SPCE-1 × 3 (i.e., 1 × 3 array).

The signal-to-noise ratio (SNR) is defined as the ratio of the power of the desired signal to the power of the undesired signal. Figure 12 shows a logarithmic relationship (R^2^ = 0.989) between the immunosensor’s SNR and the array configuration. It is seen that the immunosensor’s SNR increases logarithmically as the array configuration increases in size. This logarithmic increase means that there is a limit to the amount by which the array configuration can be used to increase the immunosensor’s SNR. Results show that ULBP2-SPCE-1 × 3 immunosensor has a statistically significant (independent *t*-test: *p* value < 0.05) and higher SNR than that of ULBP2-SPCE (about 1.5-fold) immunosensors.

**Figure 13:**
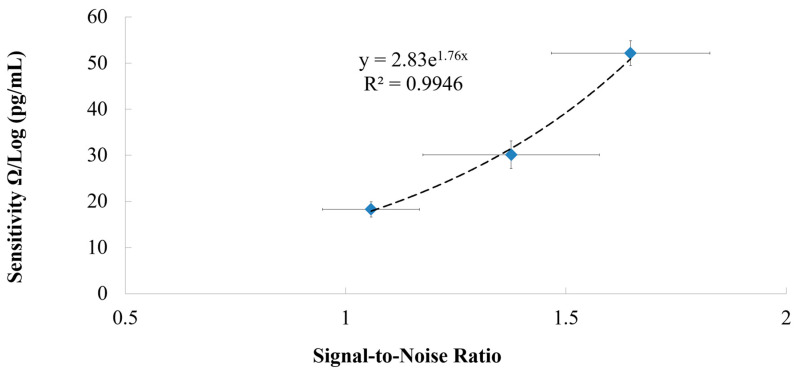
The relationship between the sensitivity and the signal-to-noise ratio of the immunosensor.

Figure 13 shows that there is an exponential relationship (R^2^ = 0.995) between the immunosensor’s sensitivity and the SNR. It is seen that the immunosensor’s sensitivity increases exponentially as the SNR increases. The array configuration can be used to increase the immunosensor’s SNR, which results in an increase in the immunosensor’s sensitivity.

### 3.5. Comparison with Other Methods for ULBP2 Antigen Detection

This study develops immunosensors for the detection of the ULBP2 antigen. Results show that the ULBP2-ZnO/SPCE immunosensor has the highest sensitivity of 332.2 Ω/Log (pg/mL) while the ULBP2-SPCE immunosensor has the lowest sensitivity of 18.5 Ω/Log (pg/mL). The descending order of the immunosensors’ sensitivity is ULBP2-ZnO/SPCE, ULBP2-SPCE-1 × 3, ULBP2-SPCE-1 × 2, and ULBP2-SPCE. On the other hand, the LOD of the ULBP2-ZnO/SPCE immunosensor is the lowest (1 pg/mL) among other immunosensors. Therefore, in this study, the best immunosensor for detecting the ULBP2 antigen is the ULBP2-ZnO/SPCE immunosensor.

Recently, Chang et al. [24] developed a high-throughput biosensor based on a two-frequency laser beam in conjunction with a 96-well immunoplate for the determination of the ULBP2 antigen. This biosensor uses metal-enhanced fluorescence and harmonic intensity-modulated fluorescence. However, the biosensor requires expensive optical laser equipment, so the ULBP2 determination must be performed at a clinic or hospital. It does not allow PC screening at home, as few people can afford the expensive optical laser equipment. The ULBP2-ZnO/SPCE immunosensor that is proposed in this study uses impedance measurement at the optimal measurement frequency (5.92 MHz). It is very cheap to manufacture an impedance readout device at 5.92 MHz, so the determination of ULBP2 for PC screening can be performed at home. 

Moreover, the proposed ULBP2-ZnO/SPCE immunosensor has a lower LOD (1 pg/mL) than Chang’s biosensor (LOD: 16–18 pg/mL). The LOD for the proposed ULBP2-ZnO/SPCE immunosensor for the determination of ULBP2 antigen is 16-fold to 18-fold better than that for Chang’s biosensor. 

A summary of the commercialized enzyme-linked immunosorbent assay (ELISA) kits to detects the human ULBP2 limit of detection can be found in Table 4. By comparing the LOD of the ELISA kits and our biosensor, the LOD is significantly improved by at least 10-fold, we can say that the developed ULBP2-SPCE/ZnO biosensor is a promising tool for future research.

## 4. Conclusions

This study developed a simple, reliable, and inexpensive immunosensor for the determination of the ULBP2 antigen. The proposed ULBP2-ZnO/SPCE immunosensor has a low LOD, high sensitivity, good repeatability, and it is stable in long-term storage. In an array configuration, the immunosensor has a great sensitivity and SNR.

## Figures and Tables

**Table 1:** Electrochemical biosensors for the detection of PC biomarkers.

Detection Method/Recognizing Elements	Biomarker	Limit of Detection	Sensitivity	Reference
Impedance spectroscopy Polysaccharide chitosan and the protein concanavalin A-Ab	CA 19-9	0.69 U/mL	n/a	[30]
Impedance spectroscopy Au-anti CA 19-9	CA 19-9	0.68 U/mL	n/a	[31]
Capacitance measurement SPE/carbon nano-onion/GO	CA 19-9	0.12 U/mL	0.23 μF log[CA19-9]−1	[32]
Impedance spectroscopy nanofiber/MWCNT-Ab nanofiber/Au-Ab	CA 19-9	1.84 U/mL with nanofiber/MWCNT-Ab 1.57 U/mL with nanofiber/Au-Ab	n/a	[33]
ELISA, high-throughput localized surface plasmon coupled harmonic fluorescence biosensor	ULBP2	16–18 pg/mL	n/a	[24]

**Table 2:** EDS showing the composition of the graphite sensor window for the SPCE.

Element	Weight %	Atomic %
C	93.99	95.42
O	6.01	4.58
Totals	100.00	

**Table 3:** Repeatability tests for the ULBP2-SPCE and ULBP2-ZnO/SPCE immunosensors.

Test		Outcome	
		*ULBP2-SPCE*	*ULBP2-ZnO/SPCE*
Repeatability	Coefficients of variation	4.55%	5.03%
	Intrarater (ICC 3,k)	0.91	0.90
	Interrater (ICC 2,k)	0.90	0.88

Remark: The evaluations were conducted by measuring the impedance response of the ULBP2-SPCE and ULBP2-ZnO/SPCE immunosensors to the ULBP2 antigen (10 pg/mL) at the optimal measurement frequency of 8.37 kHz and 5.92 MHz, respectively.

**Table 4:** ELISA Kit for the detection of ULBP2 in humans.

ULBP2 ELISA Kit (Human) ID	Kit Duration	Limit of Detection	Datasheet
OKEH01599	~3 h	34 pg/mL	[38]
OKDD07138	1–3.5 h	0.055 ng/mL	[39]
OKBB01116	~3 h	<12 pg/mL	[40]
OKCD09332	~3 h	<0.055 ng/mL	[41]
OKRC01005	n/a	13 pg/ml	[42]
SEL882Hu	~3 h	0.055 ng/mL	[43]
HUFI01846	n/a	<7.5 IU/ml	[44]

## References

[1] Global Burden of Disease Cancer Collaboration (2015). The Global Burden of Cancer 2013. JAMA Oncol..

[2] Jemal A., Siegel R., Xu J., Ward E. (2010). Cancer statistics. CA Cancer J. Clin..

[3] Pliarchopoulou K., Pectasides D. (2009). Pancreatic cancer: Current and future treatment strategies. Cancer Treat. Rev..

[4] Ministry of Health and Welfare, Taiwan (2014). 2014 statistics of causes of death. http://www.mohw.gov.tw/lp-3266-2.html.

[5] Chan A., Diamandis E.P., Blasutig I.M. (2013). Strategies for discovering novel pancreatic cancer biomarkers. J. Proteom..

[6] Carpelan-Holmström M., Louhimo J., Stenman U.H., Alfthan H., Haglund C. (2002). CEA, CA 19-9 and CA 72-4 improve the diagnostic accuracy in gastrointestinal cancers. Anticancer. Res..

[7] Kamisawa T., Wood L.D., Itoi T., Takaori K. (2016). Pancreatic cancer. Lancet.

[8] Tanaka S., Takakura R., Ioka T., Nakao M., Fukuda J., Suzuki R., Ueda E., Yoshioka F., Ashida R., Arimoto N. (2012). Detectability of high-risk signs of pancreatic cancer (pancreatic cysts and main pancreatic duct dilatation): Ultrasonography versus low-dose plain X-ray CT. Choonpa Igaku.

[9] Buscail L., Pagès P., Berthélemy P., Fourtanier G., Frexinos J., Escourrou J. (1999). Role of EUS in the management of pancreatic and ampullary carcinoma: A prospective study assessing resectability and prognosis. Gastrointest. Endosc..

[10] Bronstein Y.L., Loyer E.M., Kaur H., Choi H., David C., DuBrow R.A., Broemeling L.D., Cleary K.R., Charnsangavej C. (2004). Detection of small pancreatic tumors with multiphasic helical CT. AJR Am. J. Roentgenol..

[11] Goonetilleke K.S., Siriwardena A.K. (2007). Systematic review of carbohydrate antigen (CA 19-9) as a biochemical marker in the diagnosis of pancreatic cancer. Eur. J. Surg Oncol..

[12] Brower V. (2011). Biomarkers: Portents of malignancy. Nature.

[13] Ballesta A.M., Molina R., Filella X., Jo J., Giménez N. (1995). Carcinoembryonic antigen in staging and follow-up of patients with solid tumors. Tumour Biol..

[14] Berinstein N.L. (2002). Carcinoembryonic antigen as a target for therapeutic anticancer vaccines: A review. J. Clin. Oncol..

[15] Bezabeh T., Ijare O.B., Albiin N., Arnelo U., Lindberg B., Smith I.C. (2009). Detection and quantification of D-glucuronic acid in human bile using 1H NMR spectroscopy: Relevance to the diagnosis of pancreatic cancer. Magn. Reson. Mater. Phys. Biol. Med..

[16] Chang Y.T., Wu C.C., Shyr Y.M., Chen T.C., Hwang T.L., Yeh T.S., Chang K.P., Liu H.P., Liu Y.L., Tsai M.H. (2011). Secretome-based identification of ULBP2 as a novel serum marker for pancreatic cancer detection. PLoS ONE.

[17] Duffy M.J. (1998). CA 19-9 as a marker for gastrointestinal cancers: A review. Ann. Clin. Biochem..

[18] Steinberg W. (1990). The clinical utility of the CA 19-9 tumor-associated antigen. Am. J. Gastroenterol..

[19] Lamerz R. (1999). Role of tumour markers, cytogenetics. Ann. Oncol..

[20] Hawes R.H. (2002). Diagnostic and therapeutic uses of ERCP in pancreatic and biliary tract malignancies. Gastrointest. Endosc..

[21] Paschen A., Sucker A., Hill B., Moll I., Zapatka M., Nguyen X.D., Sim G.C., Gutmann I., Hassel J., Becker J.C. (2009). Differential clinical significance of individual NKG2D ligands in melanoma: Soluble ULBP2 as an indicator of poor prognosis superior to S100B. Clin. Cancer Res..

[22] Waldhauer I., Steinle A. (2006). Proteolytic release of soluble UL16-binding protein 2 from tumor cells. Cancer Res..

[23] Linkov F., Gu Y., Arslan A.A., Liu M., Shore R.E., Velikokhatnaya L., Koenig K.L., Toniolo P., Marrangoni A., Yurkovetsky Z. (2009). Reliability of tumor markers, chemokines, and metastasis-related molecules in serum. Eur. Cytokine Netw..

[24] Chang Y.F., Yu J.S., Chang Y.T., Su L.C., Wu C.C., Chang Y.S., Lai C.S., Chou C. (2013). The utility of a high-throughput scanning biosensor in the detection of the pancreatic cancer marker ULBP2. Biosens. Bioelectron..

[25] Zhang Q., Chen X., Tang Y., Ge L., Guo B., Yao C. (2014). Amperometric carbohydrate antigen 19-9 immunosensor based on three dimensional ordered macroporous magnetic Au film coupling direct electrochemistry of horseradish peroxidase. Anal. Chim. Acta.

[26] Rong Q., Feng F., Ma Z. (2016). Metal ions doped chitosan–poly (acrylic acid) nanospheres: Synthesis and their application in simultaneously electrochemical detection of four markers of pancreatic cancer. Biosens. Bioelectron..

[27] Indrayanto G., Brittain H.G. (2018). Validation of chromatographic methods of analysis: Application for drugs that are derived from herbs. Profiles of Drugs Substances, Excipients and Related Methodology.

[28] Şengül Ü. (2016). Comparing determination methods of detection and quantification limits for aflatoxin analysis in hazelnut. J. Food. Drug Anal..

[29] Chen N., Li W., Wu S., Zhu Y. (2018). Fluorimetric detection of reserpine in mouse serum through online post-column electrochemical derivatization. R. Soc. Open Sci..

[30] Soares A.C., Soares J.C., Shimizu F.M., Melendez M.E., Carvalho A.L., Oliveira O.N. (2015). Controlled film architectures to detect a biomarker for pancreatic cancer using impedance spectroscopy. ACS Appl. Mater. Interfaces.

[31] Soares A.C., Soares J.C., Shimizu F.M., da Cruz Rodrigues V., Awan I.T., Melendez M.E., Piazzetta M.H.O., Gobbi A.L., Reis R.M., Fregnani J.H.T.G. (2018). A simple architecture with self-assembled monolayers to build immunosensors for detecting the pancreatic cancer biomarker CA19-9. Analyst.

[32] Ibáñez-Redín G., Furuta R.H., Wilson D., Shimizu F.M., Materon E.M., Arantes L.M.R.B., Melendez M.E., Carvalho A.L., Reis R.M., Chaur M.N. (2019). Screen-printed interdigitated electrodes modified with nanostructured carbon nano-onion films for detecting the cancer biomarker CA19-9. Mater. Sci. Eng. C.

[33] Soares J.C., Iwaki L.E., Soares A.C., Rodrigues V.C., Melendez M.E., Fregnani J.H.T.G., Reis R.M., Carvalho A.L., Correa D.S., Oliveira O.N. (2017). Immunosensor for pancreatic cancer based on electrospun nanofibers coated with carbon nanotubes or gold nanoparticles. ACS Omega.

[34] Ching C.T., Sun T.P., Huang S.H., Shieh H.L., Chen C.Y. (2010). A mediated glucose biosensor incorporated with reverse iontophoresis function for noninvasive glucose monitoring. Ann. Biomed. Eng..

[35] Ching C.T., Van Hieu N., Cheng T.Y., Fu L.S., Sun T.P., Liu M.Y., Huang S.H., Yao Y.D. (2015). Liver Cancer Detection by a Simple, Inexpensive and Effective Immunosensor with Zinc Oxide Nanoparticles. Sensors.

[36] Chen Z.J., Ou X.M., Tang F.Q., Jiang L. (1996). Effect of nanometer particles on the adsorbability and enzymatic activity of glucose oxidase. Colloids Surf. B Biointerfaces.

[37] Portney L.G., Watkins M.P. (2008). Foundations of Clinical Research: Applications to Practice.

[38] Human ULBP2 ELISA detection kit (OKEH01599) Datasheet. https://www.avivasysbio.com/ulbp2-elisa-kit-human-96-wells-okeh01599.html.

[39] Human ULBP2 ELISA detection kit (OKDD07138) Datasheet. https://www.avivasysbio.com/ulbp2-elisa-kit-human-okdd07138.html.

[40] Human ULBP2 ELISA detection kit (OKBB01116) Datasheet. https://www.avivasysbio.com/ulbp2-elisa-kit-human-okbb01116.html.

[41] Human ULBP2 ELISA detection kit (OKCD09332) Datasheet. https://www.avivasysbio.com/ulbp2-elisa-kit-human-okcd09332.html.

[42] Human ULBP2 ELISA detection kit (OKRC01005) Datasheet. https://www.avivasysbio.com/ulbp-2-alcan-alpha-n2dl2-elisa-kit-human-okrc01005.html.

[43] Human ULBP2 ELISA detection kit (E80882Hu) Datasheet. http://www.cloud-clone.com/manual/ELISA-Kit-for-UL16-Binding-Brotein-2-(ULBP2)-E80882Hu.pdf.

[44] Human ULBP2 ELISA detection kit (HUFI01846) Datasheet. https://www.medical-supply.ie/product/human-ulbp2-elisa-kit.

